# The NF-κB-HE4 axis: A novel regulator of HE4 secretion in ovarian cancer

**DOI:** 10.1371/journal.pone.0314564

**Published:** 2024-12-02

**Authors:** Kyukwang Kim, Negar Khazan, Jamie L. McDowell, Cameron W. A. Snyder, John P. Miller, Rakesh K. Singh, Michelle E. Whittum, Rachael Turner, Richard G. Moore

**Affiliations:** 1 Department of Obstetrics and Gynecology, Division of Gynecologic Oncology, Wilmot Cancer Institute, University of Rochester Medical Center, Rochester, NY, United States of America; 2 Department of Microbiology and Immunology, University of Rochester, Rochester, NY, United States of America; University of South Carolina, UNITED STATES OF AMERICA

## Abstract

Ovarian cancer is the leading cause of death among gynecologic malignancies. Despite recent advancements in targeted therapies such as PARP inhibitors, recurrence is common and frequently resistant to existing therapies. A powerful diagnostic tool, coupled with a comprehensive understanding of its implications, is crucial. HE4, a clinical serum biomarker for ovarian cancer, has shown efficacy in monitoring malignant phenotypes, yet little is known about its biological role and regulatory mechanisms. Our research demonstrates that HE4 expression in ovarian cancer can be regulated by the NF-κB signaling pathway. We found that the activation of NF-κB signaling by tumor necrosis factor (TNF)-α, a cytokine found in ovarian cancer tumors and ascites, enhanced the secretion of HE4 while its inhibition suppressed HE4 levels. Nuclear translocation of the NF-κB component p65 was found to be critical for HE4 expression; induced NF-κB activation through p65 expression or constitutive IKK2 activity elevated HE4 expression, while p65 knockdown had the opposite effect. Furthermore, we observed that NF-κB mediated HE4 expression at the transcriptional level. Our data also suggests that there is a regulatory role for HE4 in the expression of α_5_-Integrin, a crucial adhesion molecule in ovarian cancer metastasis; HE4 knockdown corresponded with reduced α_5_-Integrin expression, cell migration and cell adhesion to fibronectin.

## Introduction

Ovarian cancer is the leading cause of death among gynecologic cancers, with 19,680 new cases and 12,730 deaths projected for 2024 [1]. There are multiple histologic subtypes of ovarian cancer, but the most common is epithelial ovarian cancer (EOC), which represents over 90% of cases and is very aggressive in nature [2]. Due to its vague symptomology and lack of screening tools, most patients are diagnosed at a late stage when disease is advanced [3]. Long term prognosis is poor, with a 5-year overall survival of 50% for all stages, and 30% in patients with advanced disease [1]. Initially, up to 80% of patients have an excellent response to upfront treatment with platinum and taxane based chemotherapy regimens in combination with cytoreductive surgery, however over 80% of patients will experience disease recurrence, which is usually incurable [3,4]. When disease recurs within six months of primary treatment, it is considered platinum-resistant, and subsequent treatment yields a low response [4]. In the last ten years, the incidence and death from ovarian cancer have decreased at a rate of 2.4% per year [1]. This is in part due to a growing understanding of tumor biology and the development of targeted therapies [2,3]. One notable recent innovation was the discovery of poly ADP-ribose polymerase (PARP) inhibitors, a targeted therapy for tumors with homologous recombination deficiency (HRD). While up to 50% of patients meet criteria for use of this medication, there is still a need for targeted therapies in HRD negative patients [3,4].

Human epididymis protein 4 (HE4), a protein overexpressed in ovarian cancer [5], is a potential therapeutic target for the treatment of ovarian cancer. Presently, it is used alongside CA125 as a biomarker to aid in the diagnosis of ovarian cancer, and its overexpression correlates with the presence of more aggressive disease [6]. This secretory glycoprotein was first identified in the epithelium of the distal epididymis and is a member of the whey acidic protein four-disulfide core domain (WFDC) family. It is encoded by the WFDC2 gene located on chromosome 20q13, which also contains 13 other WFDC genes, including Eppin, Elafin and SLPI [7]. WFDC family proteins are characterized by one or more WFDC domains, which contain eight highly conserved cysteine residues, forming four disulfide bonds. The WFDC family of proteins exhibit various biological functions including anti-protease, anti-viral, anti-bacterial, anti-inflammatory and cell migration properties [7,8]. The biological role of HE4 remains unclear but is hypothesized to play a role in male fertility and innate immunity [9,10]. In ovarian cancer, in vivo and in vitro studies suggest that HE4 contributes to tumor growth, metastasis, and chemoresistance [11]; however, its definitive role and regulation remain unknown.

A possible candidate for the regulation of HE4 in ovarian cancer is the NF-κB signaling pathway. NF-κB is a key transcriptional factor in cancer, typically existing as a heterodimer of p65 and p50 [12]. The NF-κB family consists of five members: p65(RelA), V-Rel reticuloendotheliosis viral oncogene homolog B(RelB), c-Rel, NF-κB1 (p105/p50), and NF-κB2 (p100/p52), which share a conserved N-terminal Rel homology domain (RHD), responsible for their dimerization and DNA binding [13]. Only p65, c-Rel, and RelB possess the transcriptional activation domain (TAD) at their C-termini required for transcriptional activity, while p50 and p52 do not [14]. p50 and p52 are derived from precursors p105 and p100, respectively, by proteasomes. Upon cytokine stimulation, the canonical pathway promotes the phosphorylation of IKK2, a component of the IκB kinase complex, leading to phosphorylation and proteasomal degradation of IκBα, which sequesters NF-κB in the cytoplasm. Consequently, NF-κB is released from IκBα and translocates into the nucleus for target gene activation. Multiple components of this signaling pathway play a crucial role in ovarian cancer progression, promoting chemoresistance, metastasis, immune evasion, and resistance to cell death [15]. Upregulation of p65 and p50 has been observed in ovarian malignancies [16]; p50 and IKK2 are associated with poor survival [17,18] and IKK2 was found to mediate aggressiveness [18]. Another IKK isoform, IKK-ε, is also frequently overexpressed in ovarian cancer [19] and has been linked to chemoresistance and metastasis [19,20]. Additionally, upstream regulators of NF-κB, such as toll-like receptor and MyD88, have been linked to an unfavorable prognosis and a more aggressive phenotype [21].

The activity of NF-κB is affected by a variety of extracellular stimuli, such as the TNF cytokine family [22]. TNF is widely expressed in ovarian cancer and studies show levels of TNFα are elevated in malignant ovarian tissues [23], serum and ascites [24]. Additionally, there is evidence that TNF levels correlate with tumor grade [25]. Inflammatory cytokines like TNF, along with NF-κB signaling activation, may play a role in regulating biomarker expression in ovarian cancer. While some studies have found that NF-κB regulates MUC16, a precursor to CA125 [26,27], little is known about HE4. In this paper we investigated whether TNF and other NF-κB activating factors affect the secretion of HE4 in ovarian cancer. We found that TNFα promotes HE4 secretion via the NF-κB signaling pathway, potentially creating a NF-κB/TNFα positive feedback loop.

## Materials and methods

Cell culture and materials: The cell lines A2780 (DMEM), SKOV-3 (DMEM), OVCAR-8 (DMEM), IGROV-1 (DMEM), RMG-1 (RPMI), 2008 (RPMI), OVCAR-3 (DMEM), HCH-1 (RPMI), 2008 (RPMI), ES-2 (McCoy’s 5A), and Caov-3 (DMEM) were obtained as previously described [28]. Each cell line was maintained at 37°C with 5% CO_2_ in its respective medium, as indicated in brackets, supplemented with 10% fetal bovine serum (FBS). Other materials were obtained from venders as follows; cytokines: TNFα (R&D Systems cat. No. 210-TA-020), IL-1β (BioLegend cat. No. 579402); chemicals: TPCA-1 (BioVision cat. No. 2431-5), BIRB-769 (APExBIO cat. No. A5639), JNK-IN-8 (APExBIO cat. No. A3520), CI-1040 (Selleckchem cat. No. S1020); siRNAs: Silencer Select siRNAs (ThermoFisher) for control (cat. No. 4390843) and HE4 (ID No. s20356). siGENOME siRNAs (horizon) for control (cat. No. D-001210-03-05), HE4 (cat. No. M-010564-02), and p65 (cat. No. M-003533-02). Santa Cruz Biotechnology siRNAs for control (cat. No. sc-37007) and HE4 (cat. No. sc-43826); Vectors: empty (addgene, cat. No. 24165), p65 (addgene, cat. No. 21966), IKK2-EE (addgene, cat. No.11105); antibodies: p53 (Santa Cruz Biotechnology cat. No. sc-126), HE4 (OriGene cat. No. UM870019), p50 (BioLegend cat. No. 603901), P-c-Jun (BioLegend cat. No. 605551). All other antibodies were purchased from Cell Signaling Technology: P-p38 (cat. No. 4511), p38 (cat. No. 9212), c-Jun (cat. No. 9165), P-p44/42 (cat. No. 4370), p44/42 (cat. No. 9102), Iκβα (cat. No. 4814), α-Tubulin (cat. No. 2144), p65 (cat. No. 8242), GAPDH (cat. No. 2118), Lamin A/C (cat. No. 4777), p21 (cat. No. 2947), Cyclin E1 (cat. No. 4129), α_4_-Integrin (cat. No. 8440), α_5_-Integrin (cat. No. 4705), β_1_-integrin (cat. No. 9699), β_4_-Integrin (cat. No. 14803), E-Cadherin (cat. No. 3195), COX2 (cat. No. 12282), ICAM-1 (cat. No. 67836).

Transfection and HE4 enzyme-linked immunosorbent assay: The cells in a 96-well plate were treated with cytokines or transfected with expression vectors or siRNAs using Lipofectamine 3000 (ThermoFisher cat. No. L3000008), following the manufacturer’s protocol. Afterward, the cell culture medium was collected to measure HE4 levels, and the cells in the plate were used to determine cell viability using the MTS assay (Promega, cat. no. G3580). The MTS assay was conducted according to the manufacturer’s instructions. HE4 levels were measured using the Human HE4/WFDC2 DuoSet ELISA (R&D Systems, cat. no. DY6274-05), following the provided protocol. Unless otherwise indicated, HE4 levels were normalized to the cell population as determined by the MTS assay.

Luciferase assay: A2780 and HCH-1 cells in a 96-well white plate were transfected with the pGL4.32[luc2P/NF-κB-RE/Hygro] vector (Promega, cat. No. E849A) or the pGL2-HE4-652 construct (a gift from K. Nephew, Indiana University). Following transfection, the cells were stimulated with TNFα or IL-1β for 7 hours. Luciferase activity was then analyzed using the Steady-Glo Luciferase Assay (Promega, cat. No. E2510) according to the manufacturer’s protocol. Simultaneously, the cell population for each condition was determined via the MTS assay to normalize the luciferase assay.

PCR assay: The cells were treated as indicated. Subsequently, total RNA was extracted using TRI Reagent (Zymo Research, cat. No. R2050), followed by cDNA synthesis using the iScript cDNA Synthesis kit (Bio-Rad, cat. No. 1708891) according to the manufacturer’s protocols. PCR was performed using the following primers: for HE4 forward: CCG ACA ACC TCA AGT GCT C, reverse: CGA GCT GGG GAA AGT TAA TG; for GAPDH forward: AAT CCC ATC ACC ATC TTC C, reverse: GTC CTT CCA CGA TAC CAA AG. PCR-amplified samples after 30 cycles were analyzed on a 2% agarose gel and visualized with SYBR™ Safe DNA Gel Stain (Invitrogen, cat. No. S33102). Quantitative PCR was conducted using a CFX384 Real-Time PCR Detection System (Bio-Rad, cat. No. 1855484) with Power SYBR Green PCR Master Mix (ThermoFisher, cat. No. 4367659). The following primers were utilized: TNFα (forward: TGC ACT TTG GAG TGA TCG G, reverse: TCA GCT TGA GGG TTT GCT AC), α_5_-integrin (forward: ACC AAC AAG AGA GCC AAA GTC, reverse: TTG TAC ACA GCC TCA CAC TG) and HE4 (forward: CCG ACA ACC TCA AGT GCT G, reverse: CGA GCT GGG GAA AGT TAA TG).

Isolation of subcellular fractions and immunoblot analysis: Cytoplasmic and nuclear protein fractionation were performed using NE-PER Nuclear and Cytoplasmic Extraction Reagents (ThermoFisher, cat. No. 78833). Whole protein extracts for all other experiments were prepared using Cell Lysis Buffer (Cell Signaling Technology, cat. No. 9803). In both methods, we followed the manufacturer’s protocol with appropriate modifications. Subsequently, immunoblot analysis was conducted to assess protein expression. Briefly, protein samples were separated via gel electrophoresis using a NuPAGE 4–12% Tris-Bis gel (Invitrogen, cat. No. NP0322) with MES SDS buffer, transferred to a nitrocellulose membrane using the Mini Blot Module (Invitrogen, cat. No. B1000), and then incubated with the indicated antibodies after blocking with 5% nonfat milk in TBS-Tween 20 buffer. If necessary, membranes were stripped with OneMinute Western Blot Stripping Buffer (GM Biosciences, Cat. No. GM6001) before reprobing.

Adhesion assay: We conducted a cell adhesion assay by coating a 96-well plate with fibronectin (50 μg/mL in PBS, Santa Cruz Biotechnology, cat. No. sc-29011, 35 μL per well) or collagen I (50 μg/mL in 20 nM acetic acid, Gibco, cat. No. A10483-01, 35 μL per well) at 37°C for 1 hour, followed by two washes with 0.1% bovine serum albumin (BSA) in PBS. The plates were then blocked with 2% BSA in PBS for 1 hour. 2008 cells, transiently transfected with HE4 or control siRNA for 72 hours, were harvested by trypsinization, washed in PBS, and plated into the coated wells in RPMI medium without FBS. After incubation in a CO_2_ incubator at 37°C for 1 hour, the cells were washed twice with 0.1% BSA in PBS and fixed with 5% trichloroacetic acid for the SRB cell population assay [29]. For normalization, the same transfected cells were plated in RPMI medium with FBS and allowed to adhere to the surface before the SRB assay was conducted.

Cell Migration Assay: 2008 cells were seeded in a 12-well plate at a density of 200,000 cells per well and allowed to adhere overnight, followed by transfection with HE4 siRNA or siRNA control. After 48 hours of transfection, each well received a single vertical scratch using a P1000 tip. The scratched wells were rinsed once with culture media to remove detached cells. Initial images were taken immediately following this, represented by the “0 hr” timepoint. Images were taken of the scratch at the same location in 6-hour intervals for 18 hours. Images were taken on an Olympus CKX41 inverted phase-contrast microscope with an Olympus DP74 camera and CellSens software. Image analysis was completed using Fiji’s ImageJ software and the wound healing size tool plugin developed by Suarez-Arnedo et al. [30].

Statistical analysis: All statistical analyses were conducted using GraphPad Prism 10 software with a two-tailed unpaired t-test. Significance levels were denoted as follows: **** for p ≤ 0.0001, ** for p ≤ 0.01, and ns for not significant.

## Results

### TNFα promoted the secretion of HE4 in ovarian cancer cells

**Fig 1:**
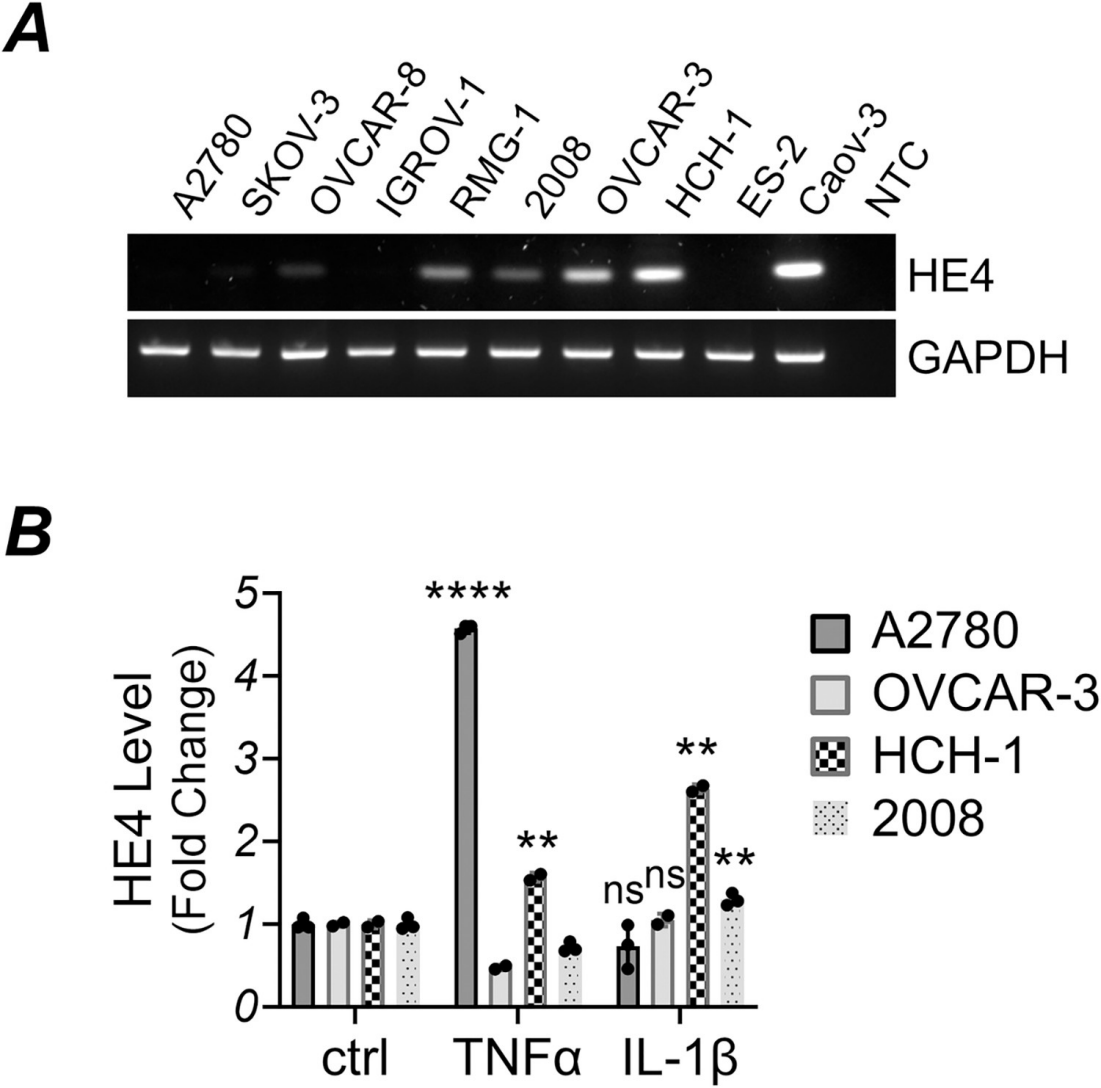
(A) Expression of HE4 in ovarian cancer cell lines. mRNA expression of HE4 was determined by semiquantitative reverse transcription PCR. GAPDH served as a loading control. NTC: Non-template control. (B) TNFα and IL-1β promote the secretion of HE4 in ovarian cancer cell lines. Cells were subjected to TNFα (30 ng/mL) or IL-1β (10 ng/mL) treatment for 72 hours. Afterward, cell culture medium was collected. HE4 levels were determined using HE4 ELISA.

Serum levels of HE4 are highly upregulated in ovarian cancer and are utilized as a clinical marker for its detection. However, HE4 levels in ovarian cancer cell lines may vary and can sometimes be undetectable [31]. The expression profile of HE4 was assessed in a panel of ovarian cancer cell lines. Semiquantitative reverse transcription PCR revealed HE4 mRNA was present in RMG-1, 2008, OVCAR-3, HCH-1, and Caov-3 cell lines, while other cell lines showed no or weak expression (Fig 1A). Next, to assess the role of NF-κB on HE4 expression, we treated OVCAR-3, 2008 and HCH-1 cells with TNFα. These cell lines were selected because they express HE4, indicating the presence of functional HE4 regulatory machinery. A cell line with weak HE4 expression, A2780, was also treated with TNFα. Following the treatment, we collected the cell culture medium and analyzed HE4 levels with enzyme-linked immunosorbent assay (ELISA). Treatment with TNFα markedly increased HE4 secretion in A2780 and HCH-1 cells (Fig 1B). Additionally, another NF-κB activating cytokine, IL-1β, promoted HE4 secretion in HCH-1 and 2008 cells. This effect appears specific to certain cell types and cytokines, as neither TNFα nor IL-1β was able to induce HE4 secretion in OVCAR-3 cells, and IL-1β did not induce HE4 secretion in A2780 cells.

### NF-κB signaling pathway mediated HE4 secretion in ovarian cancer cells

**Fig 2:**
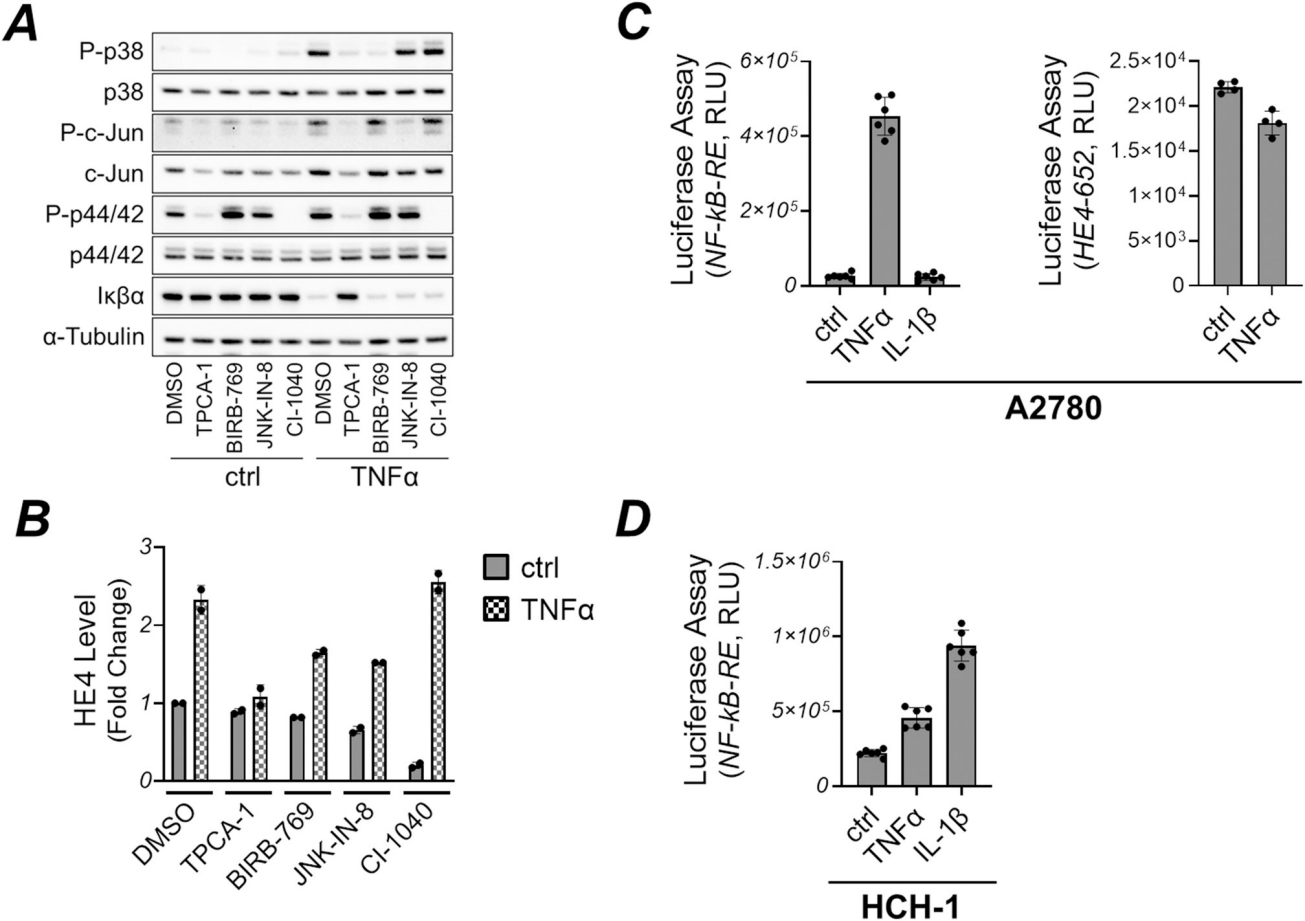
TNFα promoted HE4 secretion through the NF-kB signaling pathway (A) A2780 cells were incubated with TPCA-1 (25 μM, an IKK2 inhibitor), BIRB-769 (1 μM, a p38 inhibitor), JNK-IN-8 (1 μM, a JNK inhibitor), or CI-1040 (20 μM, a MEK inhibitor) for 1 hour, after which cells were subjected to TNFα treatment (30 ng/mL) for 15 minutes. Subsequently, protein expression and phosphorylation were assessed via immunoblot analysis using target-specific antibodies, with α-tubulin serving as a loading control. (B) A2780 cells underwent the same inhibitor treatment as in (A), after which cells were treated with TNFα (30 ng/mL) for 24 hours. HE4 levels in culture medium were determined using HE4 ELISA. (C) A2780 cells were transfected with a construct encoding luciferase under the control of an NF-κB response element or HE4 promoter construct (HE4-652), and luciferase activity was assessed following TNFα (30 ng/mL) or IL-1β (10 ng/mL) treatment for 7 hours. (D) Same as (C), but HCH-1 cells were employed.

TNFα is a multifaceted cytokine, and mediates the activation of many signaling pathways, including apoptosis, the mitogen-activated protein (MAP) kinase family (p38, JNK, and p44/42), and NF-κB [32,33]. To elucidate which pathway TNFα employs to induce HE4 expression, A2780 cells were pretreated with pathway-specific pharmacologic inhibitors—TPCA-1, BIRB-769, JNK-IN-8, and CI-1040, inhibiting NF-κB, p38, JNK, and p44/42 signaling, respectively—prior to TNFα stimulation. Subsequently, their impact on HE4 secretion was assessed. Immunoblot analysis revealed that inhibitor treatment effectively blocked each targeted signaling pathway triggered by TNFα (Fig 2A). Among these, only NF-κB inhibition by TPCA-1 markedly blocked HE4 upregulation by TNFα treatment (Fig 2B). To this end, we also assessed TNFα activity using an NF-κB-dependent transcriptional reporter assay with five copies of an NF-κB response element (RE) located upstream of luciferase. The luciferase reporter construct was transiently transfected into A2780 and HCH-1 cells. Following transfection, the cells were treated with TNFα and IL-1β, and luciferase activity was subsequently measured. We found that TNFα markedly increased luciferase activity in A2780 cells, whereas IL-1β failed to elicit a similar response (Fig 2C). Conversely, in HCH-1 cells, both TNFα and IL-1β demonstrated an increase in luciferase activity. These results are consistent with the effects of these cytokines on HE4 secretion in Fig 1B. Additionally, we transfected A2780 cells with the HE4 promoter reporter construct used in a previous study [34] and stimulated the cells with TNFα. TNFα did not increased the activity of this promoter (Fig 2C).

### Modulating NF-κB signaling components influenced HE4 expression

**Fig 3:**
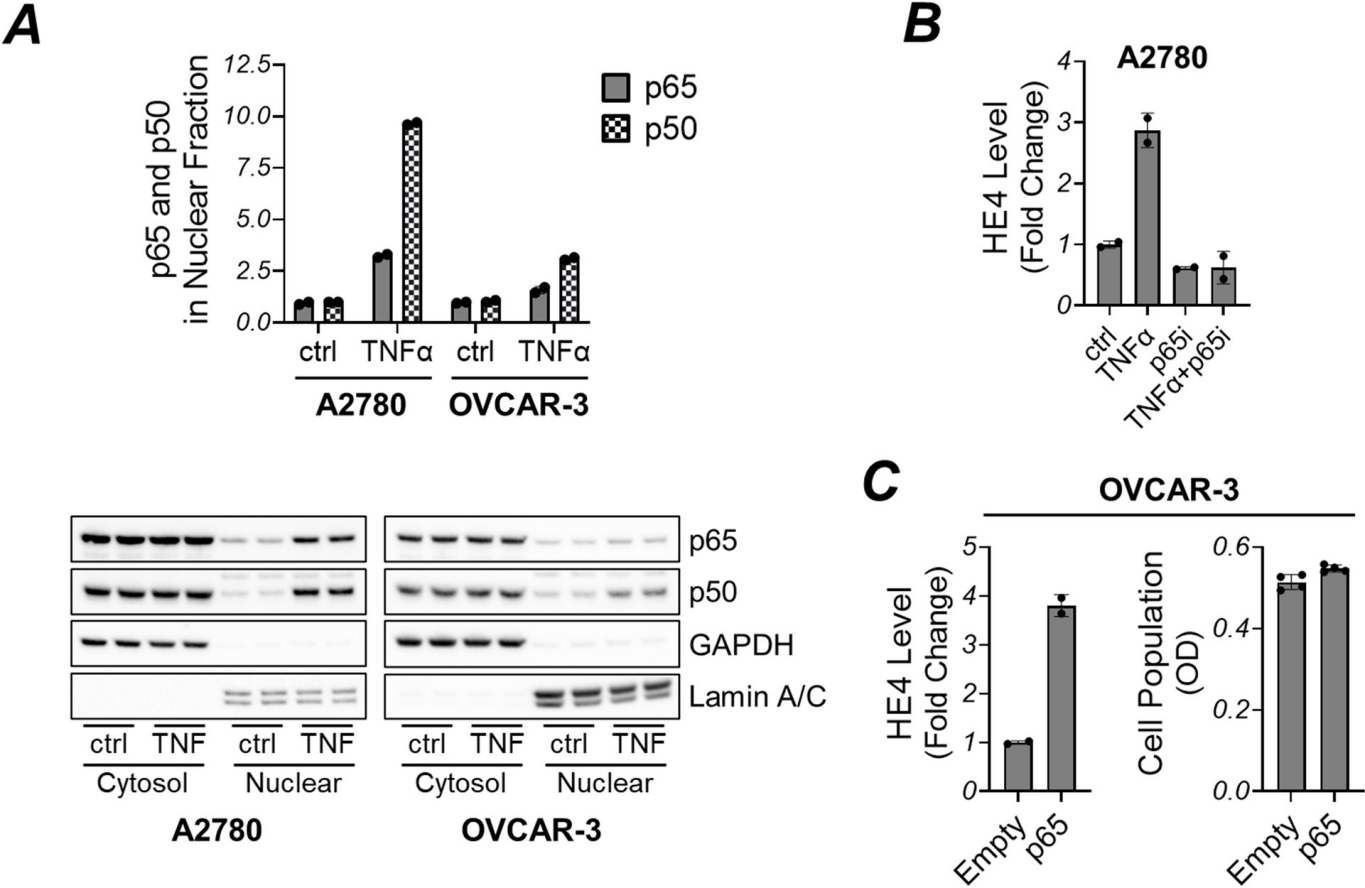
(A) TNFα enhanced the nuclear localization of p65 and p50 in A2780 cells. A2780 and OVCAR-3 cells were stimulated with TNFα (30 ng/mL) for 5 hours. Cells were lysed, and cytoplasmic and nuclear fractions were isolated, followed by immunoblot analysis using antibodies against GAPDH (a cytoplasmic marker) and lamin A/C (a nuclear marker), as well as against p65, and p50. The expression levels of p65 and p50 in nuclear fraction upon TNFα stimulation were determined relative to Lamin A/C expression (top). (B) p65 knockdown abolished TNFα induced HE4 secretion in A2780 cells. A2780 cells were transfected with siRNAs against p65 or with non-targeting control. Following transfection cells were exposed to TNFα treatment (30 ng/mL) for 48 hours. Afterward, cell culture medium was collected. HE4 levels were determined using HE4 ELISA. (C) p65 expression promotes HE4 expression in OVCAR-3 cells. Cell culture medium from OVCAR-3 cells transfected for 72 hours with empty or p65 expression vector was analyzed for HE4 levels using HE4 ELISA (left). Simultaneously, the cell population for each condition was assessed via the MTS assay (right).

**Fig 4:**
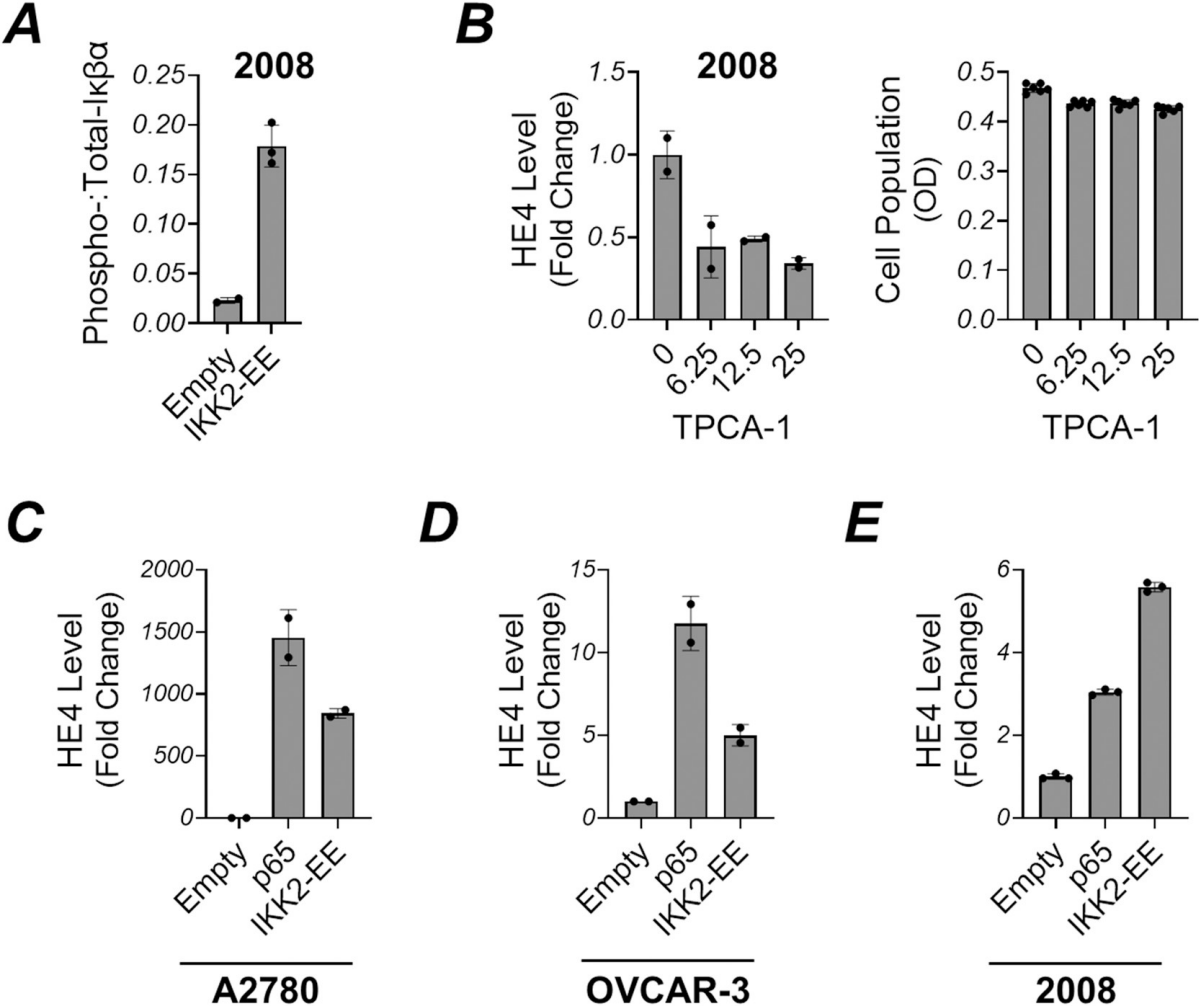
(A) Expression of constitutively active IKK2 mutant (IKK2-EE) led to the phosphorylation of Iκβα. 2008 cells were transfected with either IKK2-EE or an empty vector for 24 hours. Cells were lysed, and protein expression was assessed via immunoblot analysis. Phosphorylated Iκβα levels were quantified relative to total Iκβα. (B) IKK2 inhibitor TPCA-1 reduced HE4 expression. 2008 cells were treated with TPCA-1 at a range of concentrations for 24 hours. HE4 levels in culture medium were determined using HE4 ELISA (left). Simultaneously, the cell population for each condition was assessed via the MTS assay (right) (C-E) Expression of p65 and IKK2-EE promoted HE4 expression in ovarian cancer cell lines. A2780, OVCAR-3, and 2008 cells were transfected with p65, IKK2-EE, or empty vector for 72 hours. Afterward, HE4 levels in culture medium were determined using HE4 ELISA.

As previously noted, TNFα and IL-1β stimulation did not alter HE4 secretion in OVCAR-3 cells (Fig 1B). To investigate whether impaired NF-κB signaling in OVCAR-3 is responsible for this, we analyzed nuclear translocation of the NF-κB complex, the p65/p50 heterodimer involved in canonical NF-κB signaling. A2780 cells were also tested for comparison. In TNFα-treated A2780 cells, immunoblot analysis showed a marked elevation in nuclear p65 and p50 levels when normalized to Lamin and GAPDH, which served as loading controls for nuclear and cytosolic fractions, respectively. However, in OVCAR-3 cells, TNFα induced nuclear localization was comparatively lower (Fig 3A). Based on these findings, we carried out two further experiments: (1) knock out of p65 in A2780 cells, and (2) overexpression of p65 in OVCAR-3 cells. We then analyzed changes in HE4 expression. Our findings showed that knock down of p65 potently inhibited TNFα-induced HE4 expression in A2780 cells (Fig 3B), while overexpression of p65 in OVCAR-3 cells led to the upregulation of HE4 (Fig 3C). Next, we assessed an upstream regulator of NF-κB. The phosphorylation of IκBα by IKK2 and its degradation is an essential step for NF-κB to enter nucleus. We observed that 2008 cells exhibited a poor response in HE4 expression upon TNFα exposure and only a minor increase with IL-1β (Fig 1B). Therefore, we transiently transfected 2008 cells with an IKK2 mutant construct (IKK2-EE), designed to express constitutively active IKK2 [35]. Following transfection, IκBα phosphorylation was notably increased, leading to elevated HE4 levels in the 2008 cells (Fig 4A and 4E). The same was true for other cell lines such as A2780 and OVCAR-3 cells (Fig 4C and 4D). Additionally, we found that an IKK2 inhibitor TPCA-1 efficiently reduced HE4 levels in 2008 cells (Fig 4B).

### NF-κB activation transcriptionally controlled HE4 expression

**Fig 5:**
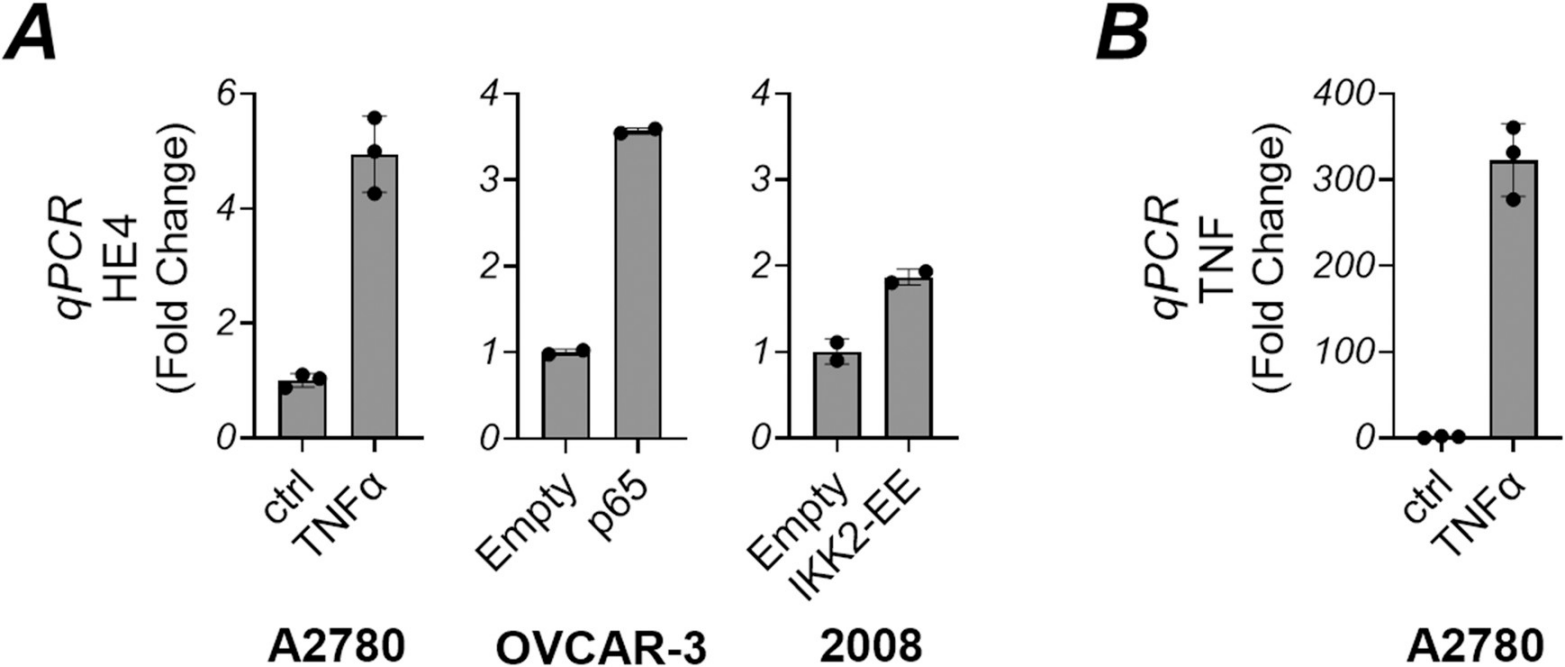
NF-kB upregulated the mRNA levels of HE4. A2780 cells were treated with TNFα (30 ng/mL) for 24 hours. OVCAR-3 and 2008 cells were transfected with p65, IKK2-EE, or empty vector for 24 hours. Cells were lysed, and isolated RNAs were analyzed for HE4 (A) or TNF (B) via quantitative reverse transcription PCR, as described in “Materials and Methods”.

Next, we interrogated whether NF-κB activation induces HE4 at the transcriptional level. A2780, OVCAR-3, and 2008 cells were either stimulated with TNFα or transfected with p65 or IKK2-EE. Subsequently, RNA was extracted, reverse transcribed into cDNA and subjected to real-time qPCR analysis. In A2780 cells, TNFα induced the upregulation of HE4 mRNA expression (Fig 5A). Moreover, transfection with either p65 or IKK2-EE, in OVCAR-3 and 2008 respectively, similarly increased HE4 at the transcriptional level, further supporting our findings. Additionally, A2780 cells exposed to TNFα markedly increased TNF mRNA expression (Fig 5B), suggesting a positive feedback loop to produce more HE4.

### HE4 knockdown reduced α_5_-integrin expression, as well as ovarian cancer cell adhesion and migration

**Fig 6:**
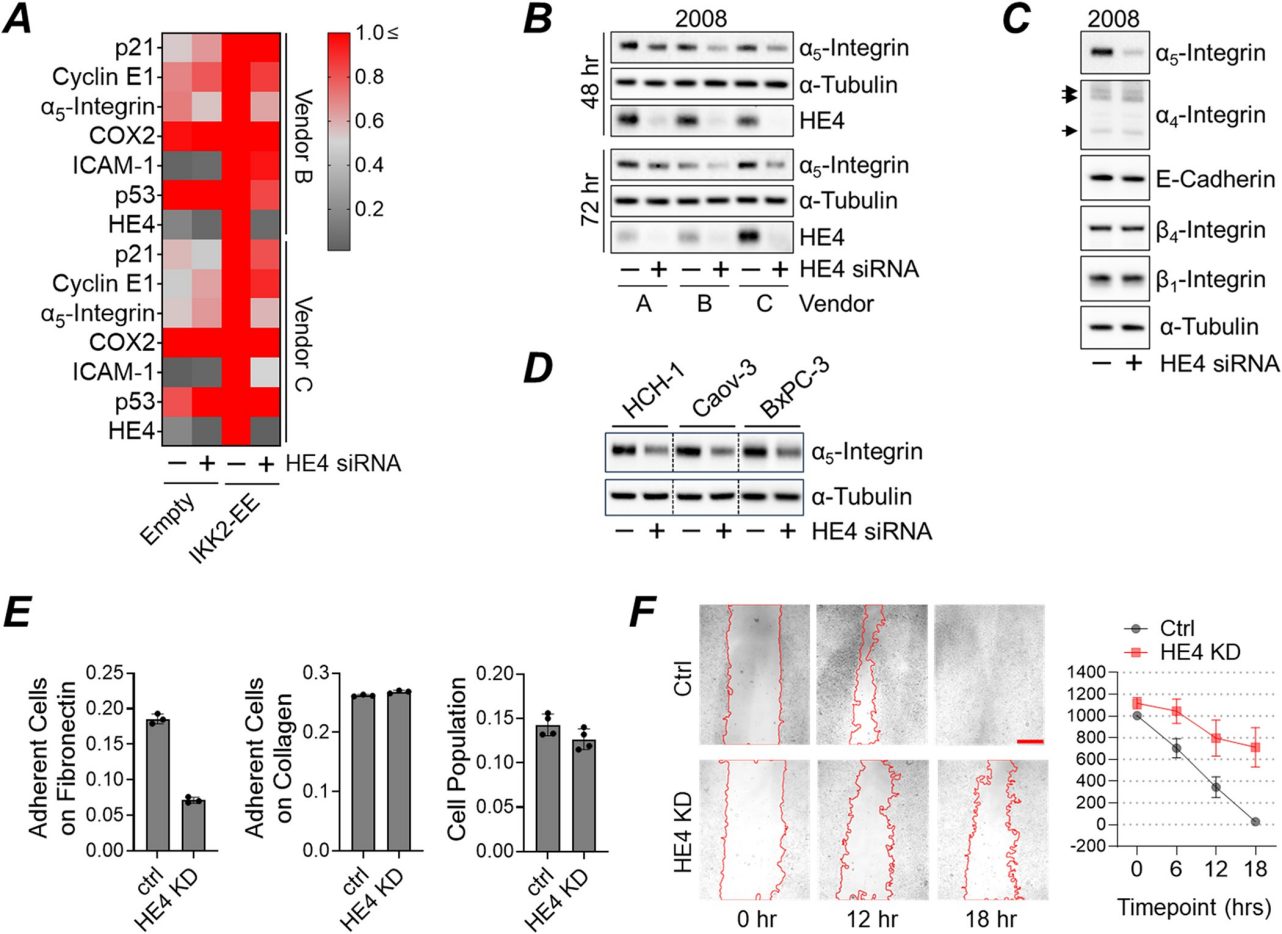
NF-kB promoted α_5_-integrin, while HE4 knockdown blocked its expression. (A) 2008 cells were co-transfected with IKK2-EE expression vector and HE4 siRNAs (sourced from two different vendors) for 48 hours. Cells were lysed, and protein expression of putative NF-kB targets was measured via immunoblot analysis. Heat map represents the relative expression of each NF-kB target evaluated by densitometric analysis. GAPDH served as a loading control for normalization. (B) 2008 cells were transfected with HE4 siRNAs from three different vendors (48 or 72 hours). Expression of HE4, α_5_-integrin, and α-tubulin was measured via immunoblot analysis. (C)The effects of HE4 knockdown on adhesion molecules. 2008 cells were transfected with HE4 siRNA. Protein extracts were prepared and subjected to immunoblot analysis against the proteins as indicated. (D) as in (C) but other ovarian (HCH-1 and Caov-3) and non-ovarian (BxPC-3, pancreatic) cancer cell lines were employed. (E) HE4 knockdown inhibited cell adhesion to fibronectin. Cell adhesion of 2008 cells transfected with HE4 siRNA (72 hours) was determined in fibronectin- or collagen-coated cell culture plate as described in “Materials and Methods.” (F) HE4 knockdown inhibited cell migration. 2008 cells were transfected with either control or HE4 siRNA and cultured until confluence. A scratch was made using a P1000 pipette tip, and images were captured at 0, 6, 12, and 18 hours (scale bar: 500 μm). Right panel: The average width of the gap (y-axis, in μm) was calculated as described in the “Materials and Methods” section.

NF-κB activation triggers downstream signaling cascades, resulting in the upregulation of cancer-related genes such as COX-2, ICAM-1, integrins, p21, p53, and cyclin E1. These genes are involved in regulating the cell cycle, inflammation, and cell adhesion [36–38]. We designed a pilot experiment to assess the role of HE4 in the regulation of NF-κB target proteins. 2008 cells were co-transfected with the IKK2-EE construct and HE4 siRNAs. Protein extracts were harvested and subjected to immunoblot analysis. Our data showed that IKK2-EE overexpression led to the upregulation of putative NF-κB targets such as p21, cyclin E1, α_5_-integrin, and ICAM-1 (Fig 6A). Interestingly, among those targets, HE4 knockdown appeared to diminish the expression of α_5_-integrin (Fig 6A). Given the pivotal role of α_5_β_1_-integrin dimer in ovarian cancer progression, particularly during peritoneal metastasis [39–41], we further investigated the impact of HE4 knockdown in 2008 cells. The cells were transfected with HE4 siRNAs obtained from three different companies and incubated for 48 or 72 hours. Immunoblot analysis showed that HE4 knockdown reduced α_5_-integrin expression as soon as 48 hours post-transfection (Fig 6B), with the effect persisting for at least 72 hours. The siRNAs from all three sources proved effective in diminishing both HE4 and α_5_-integrin expression in 2008 cells. Further investigation into other integrins revealed that while α_5_-integrin levels decreased, there was no significant alteration in the expression of α_4_-, β_1_-, or β_4_-integrins (Fig 6C). E-cadherin, the adhesion molecule recognized for its role in ovarian cancer metastasis [40], also remained unchanged following HE4 knockdown. We also tested the effects of HE4 knockdown in other ovarian cancer cell lines (HCH-1 and Caov-3) and a pancreatic cancer cell line (BxPC-3). In all cases, HE4 knockdown decreased α_5_-integrin expression (Fig 6D). α_5_β_1_-integrin is the receptor of fibronectin, a major extracellular matrix component. Disturbing their interaction proved effective in controlling malignancy [42,43]. Likewise, a function blocking α_5_-integrin antibody effectively inhibited in vivo ovarian cancer cell adhesion and peritoneal metastasis [40]. This prompted us to conduct a pilot study to assess the impact of HE4 knockdown on ovarian cancer cell adhesion and migration. We first established HE4 knockdown of 2008 cells using HE4 or non-targeting control siRNAs, plated the cells into fibronectin-coated or collagen-coated plates, and allow time to adhere. After unbound cells were removed, adherent cells were fixed and analyzed for cell populations. Our data showed that HE4 knockdown led to a 38% decrease in cell attachment on fibronectin-coated surface while it had no effect on collagen (Fig 6E). Because blocking fibronectin and α_5_β_1_-integrin interactions promoted cell death [42] we also assessed the cell population following HE4 knockdown. In 2008 cells, HE4 knockdown did not impact cell viability, as the cell populations of both the control and HE4 knockdown groups remained comparable up to 72 hours post-transfection. Lastly, we conducted a wound healing assay to measure cell migration following HE4 knockdown. 2008 cells transfected with either HE4 or control siRNA were cultured to monolayer confluency and received a single vertical scratch. The wound healing process was monitored every 6 hours. We observed that wound closure was relatively slower in HE4 knockdown cells (Fig 6F).

## Discussion

Our findings support the hypothesis that the expression of HE4, a critical biomarker and potential regulators in cancer malignancies, is controlled by the NF-κB pathway. Elevated levels of NF-κB subunits, along with overexpression of NF-κB activating cytokines, have been associated with poor prognosis in ovarian cancer [16,17,23,24,44,45]. NF-κB pathways can be triggered by various stimuli, including viral and bacterial products, cytokines, chemotherapy, radiation, growth factors, oxidative stress, and DNA damage [46,47]. In the tumor microenvironment, inflammatory cancer-associated fibroblasts, tumor-associated macrophages, as well as cytokine-rich peritoneal fluid, can contribute to extracellular NF-κB signaling [48–50]. Moreover, cancer cells themselves often display intrinsic NF-κB activity due to oncogenic mutations [46]. The complexity of NF-κB activation and signaling dynamics is further compounded by other variables such as crosstalk with other signaling pathways, feedback responses, and fluctuations in signaling molecule concentration and duration [51]. This collectively influences cancer cells’ ability to perceive, interpret, and respond to both intracellular and extracellular environmental cues, leading to downstream signal transduction and possibly the induction of HE4 expression. Previous research into the regulatory mechanisms of HE4 is very limited. One study reported that hypoxia, a factor known for NF-κB signaling activation [52], induced the expression of HE4 [53]. In contrast, estrogen, a factor that may hinder NF-κB activity, also increased HE4 expression [54–56]. Further studies will be needed to explore these mechanisms.

HE4 expression is not cancer specific [57–59]; it is also expressed in a wide range of normal human tissues, including the epididymis, female reproductive tract, salivary glands, and respiratory tract [60]. Notably, HE4 has also been found in the fallopian tube, a known origin for some ovarian carcinomas [60–62]. Because NF-κB signaling is critical for tumor initiation and progression [46], it is possible that pathoclinical conditions involving NF-κB may influence HE4 overexpression, either at the onset of oncogenic transformation or during progression to a more aggressive tumor phenotype. Our study helps to understand how HE4 distinguishes malignancies from benign or borderline lesions, both of which can express HE4 [61]. It’s plausible that NF-κB activity in these lesions is lower than that in malignancies [16,45]. There is a pressing need for further studies to define the role of NF-κB in HE4 regulation in vivo and to explore its implications in other pathological conditions such as endometriosis, where NF-κB signaling is also believed to play a role [63].

In this paper, we present evidence suggesting HE4 may play a regulatory role in the expression of α_5_-integrin, a connection previously unknown. Our preliminary data suggest that HE4 may regulate α_5_-integrin transcriptionally, as HE4 knockdown in 2008 cells downregulates α_5_-integrin mRNA levels (S1 Fig in S1 File). However, the potential for post-translational control, such as HE4-depedent protein degradation, should not be ruled out. further in-depth studies will be needed to clarify the precise mechanistic functions of HE4. Nevertheless, our findings offer valuable insights into interpretation of previous findings: overexpression of HE4 promotes cell adhesion and migration, while its knockdown inhibits cell migration [64,65]. Our study suggests that HE4-mediated regulation of α_5_-integrin may contribute to those effects. α_5_-integrin, as a component of α_5_β_1_-integrin complex, is pivotal in ovarian cancer metastasis [41]. This process is unique, characterized by peritoneal dissemination. Malignant cells detach from the primary tumor and enter the abdominal cavity where they assemble multicellular aggregates, known as spheroids, essential for enhanced cell survival. Subsequently, spheroids reattach to the peritoneum at the secondary site, migrate, and further invade the tissue [66]. α_5_-integrin appears to play a crucial role in every step of this metastatic progression [40,66,67]. Additionally, α_5_-integrin expression is considered a risk factor for chemoresistance and a poor prognostic marker in ovarian cancer [40,68]. Therefore, therapies targeting α_5_-integrin, like volociximab [69], or HE4 inhibition, could be a promising strategy to inhibit ovarian cancer progression.

In conclusion, we found that the NF-κB pathway may play a pivotal role in modulating HE4 levels. The association of poor outcomes with elevated HE4 and increased NF-κB activity hints at their involvement in disease progression. Intriguingly, the possibility that HE4 could influence α_5_-integrin expression paves the way for novel insights into the pathways of ovarian cancer metastasis and its evasion of chemotherapy. Further study is warranted and could be crucial in unraveling the intricacies of HE4 and developing targeted therapeutic interventions.

## Supporting information

S1 File HE4 knockdown reduces α5-integrin mRNA expression in 2008 cells. (DOCX)

S2 File (DOCX)

S1 Dataset (XLSX)
