# Supplementary material for: The NF-κB-HE4 axis: A novel regulator of HE4 secretion in ovarian cancer
Source: PLoS One. 2024 Dec 2;19(12):e0314564. doi: 10.1371/journal.pone.0314564 (PMC11611113; doi:10.1371/journal.pone.0314564)
Supplement: S1 File — (DOCX) [file pone.0314564.s001.docx]

The NF-κB-HE4 Axis: A Novel Regulator of HE4 Secretion in Ovarian Cancer

PLOS ONE


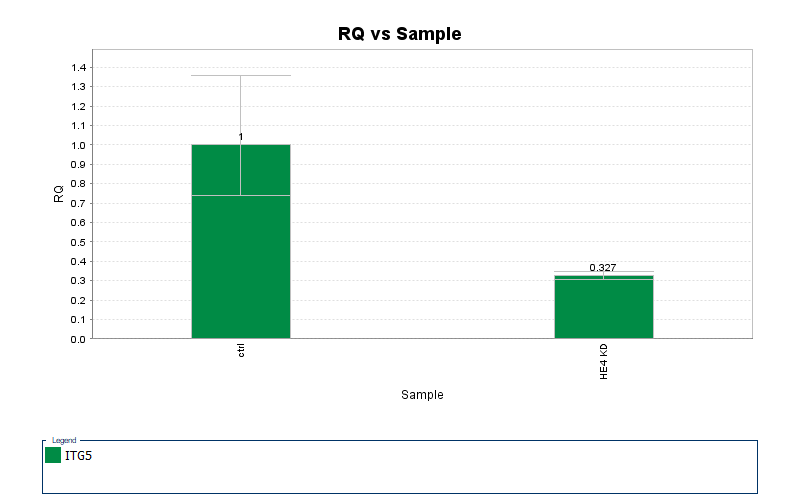


Figure S1. HE4 knockdown leads to a reduction in α_5_-integrin mRNA expression. 2008 cells were transfected with siRNA against HE4 (ThermoFisher, Silencer Select ID No. s20356) or with non-targeting control (cat. No. 4390843) for 48 hours. Cells were lysed, and isolated RNAs were analyzed for α_5_-integrin via qRT-PCR, as described in “Materials and Methods”.
