# Supplementary material for: The NF-κB-HE4 axis: A novel regulator of HE4 secretion in ovarian cancer
Source: PLoS One. 2024 Dec 2;19(12):e0314564. doi: 10.1371/journal.pone.0314564 (PMC11611113; doi:10.1371/journal.pone.0314564)

The NF-κB-HE4 Axis: A Novel Regulator of HE4 Secretion in Ovarian Cancer

PLOS ONE

Figure 2A


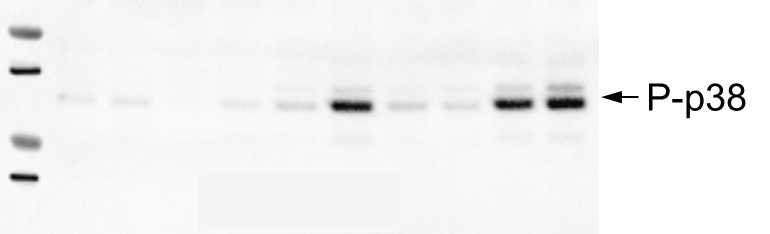


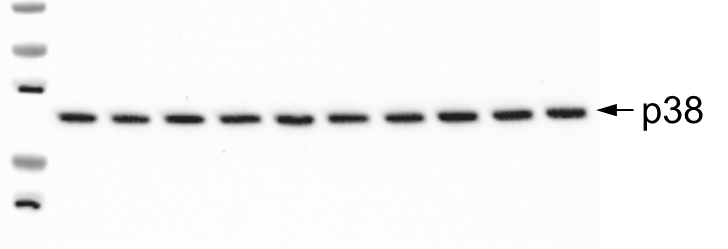


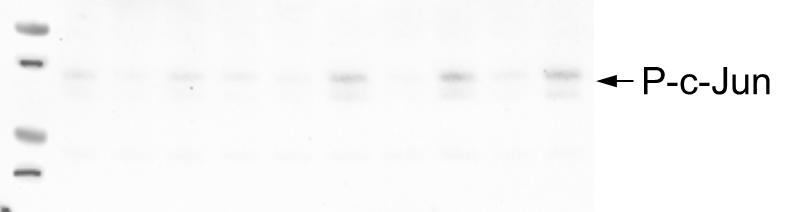


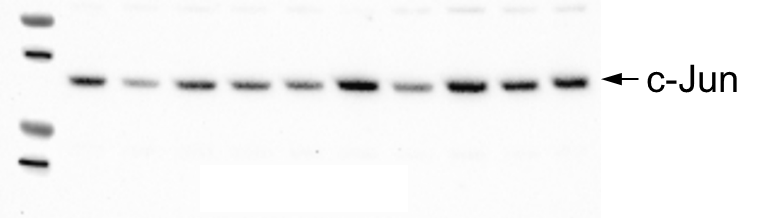


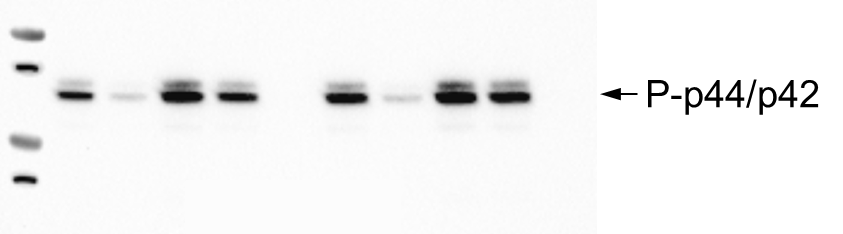


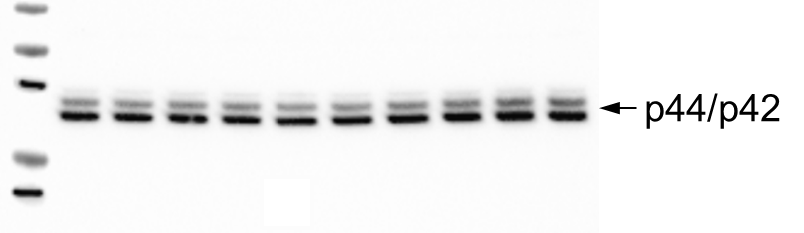


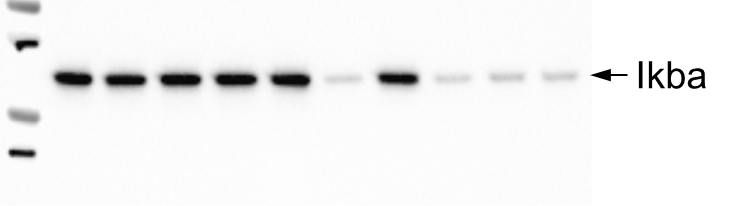


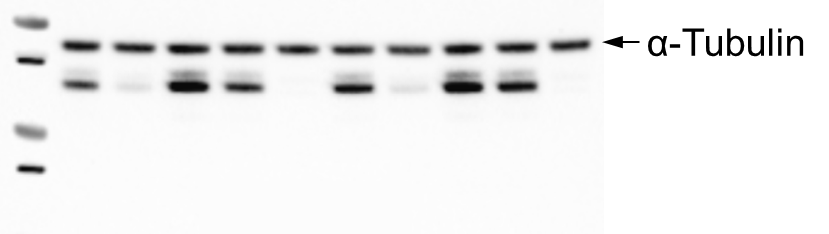


Figure 3A (left panel)


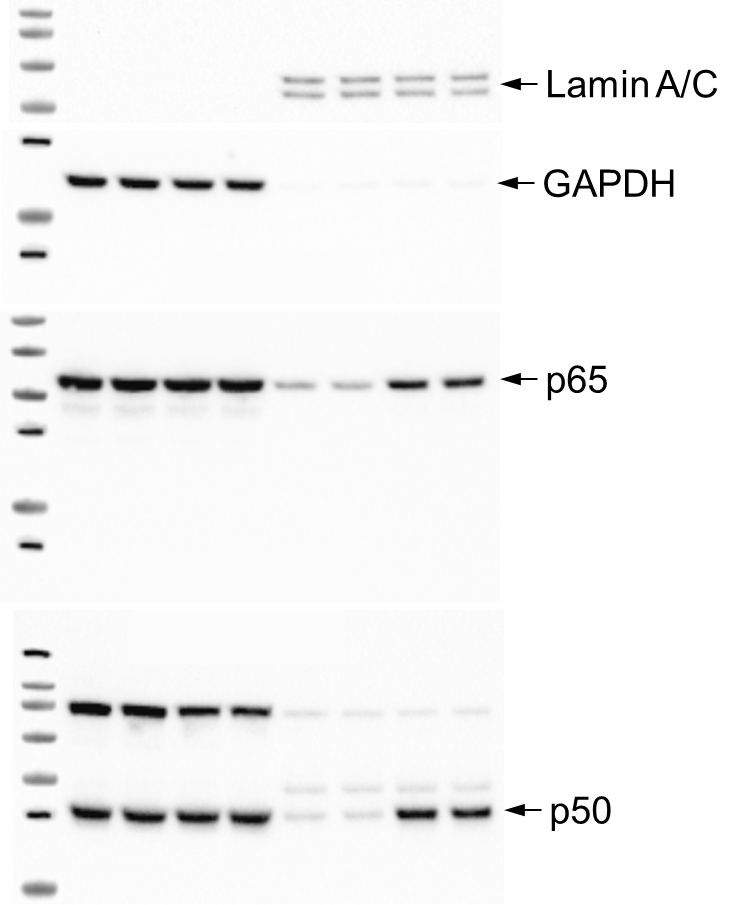


Figure 3A (right panel)


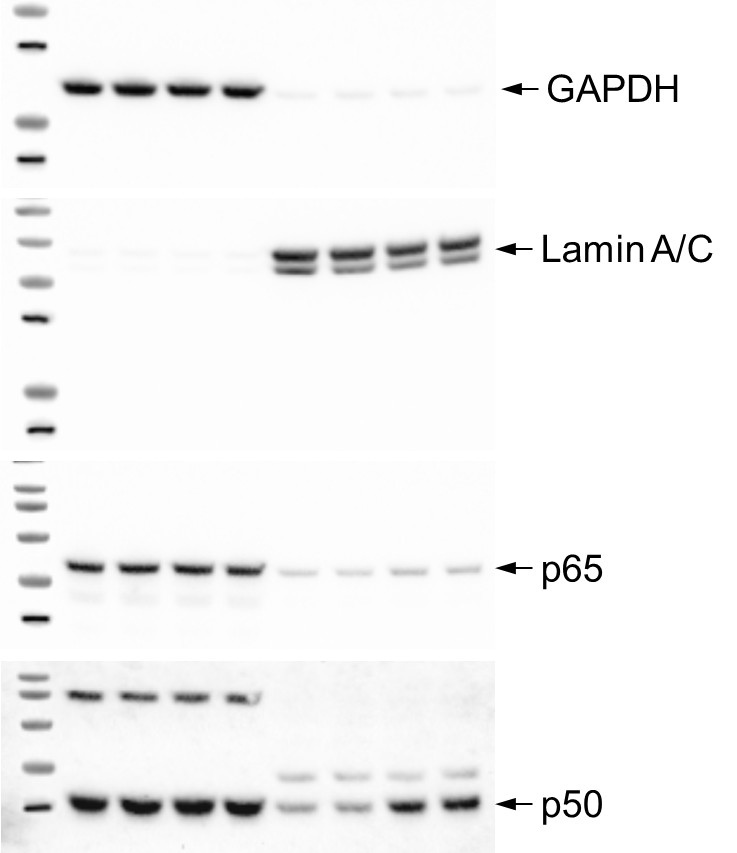


Figure 6A


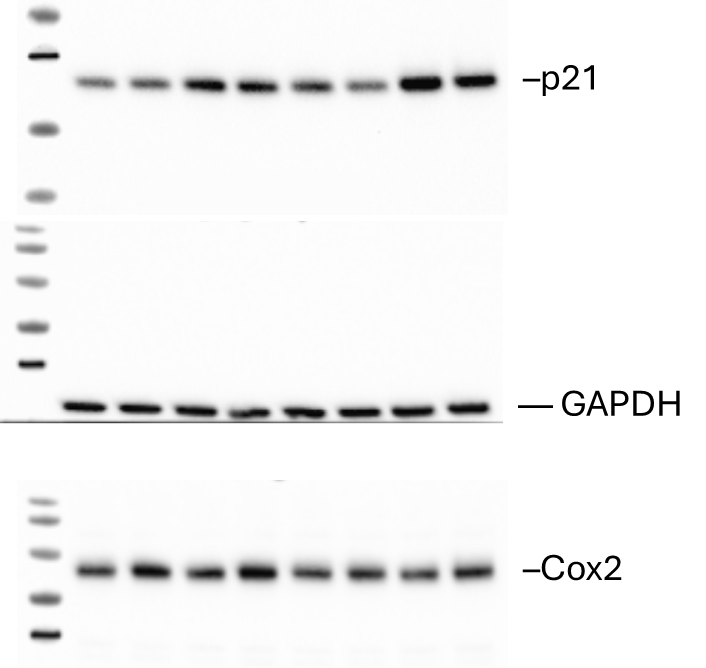


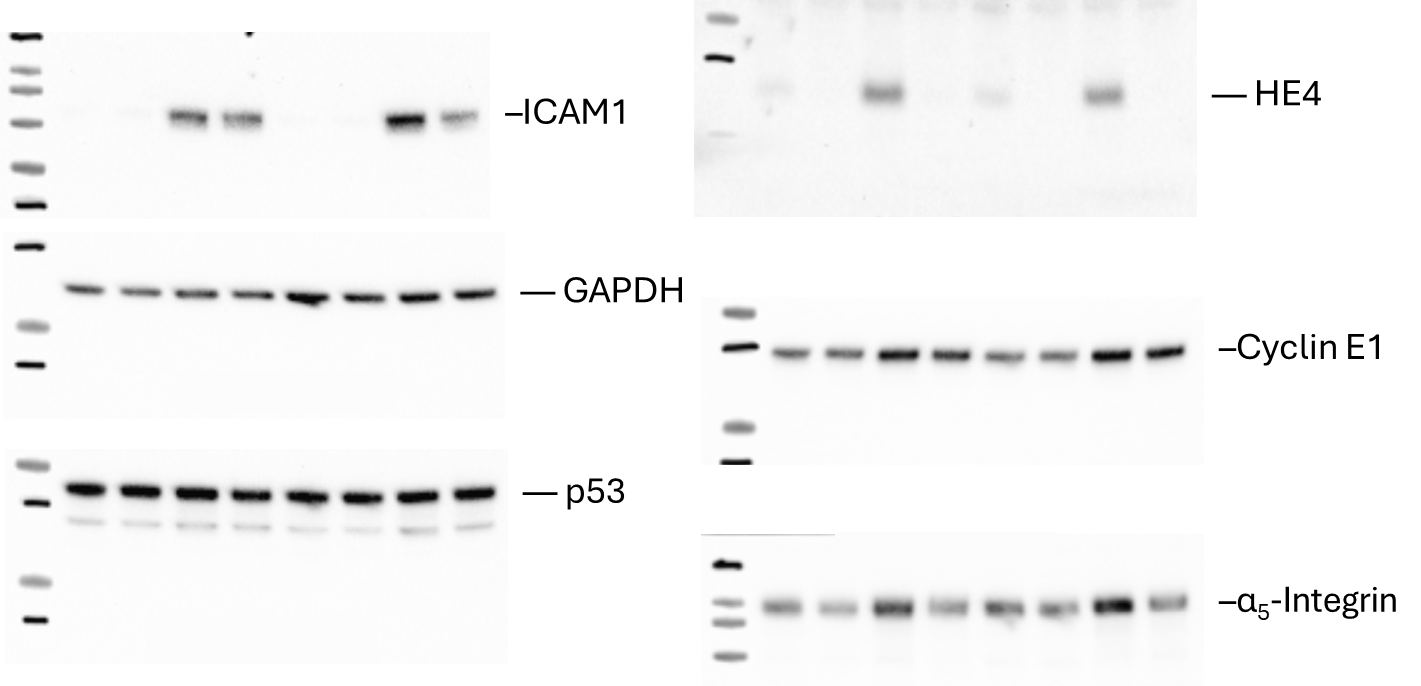


Figure 6B


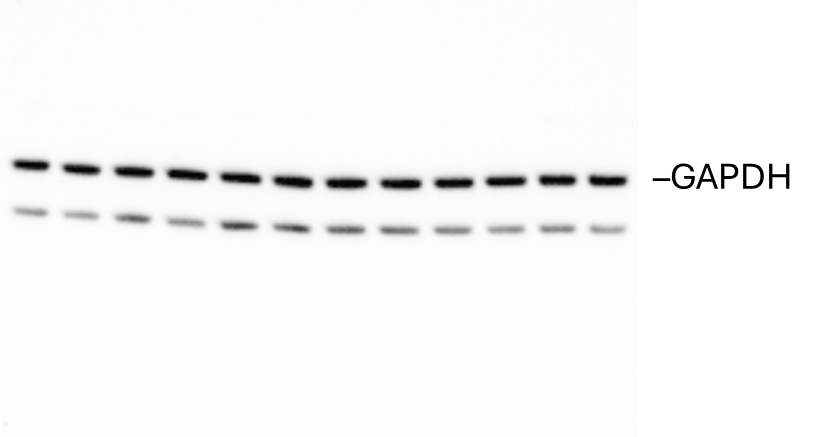


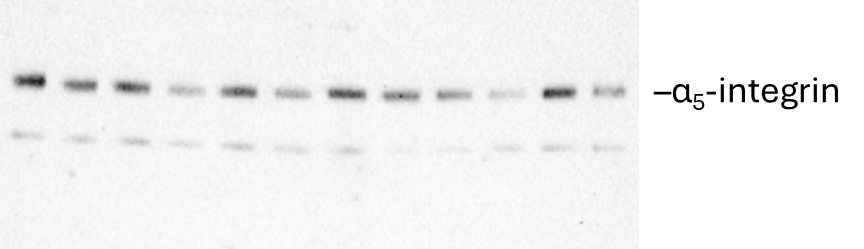


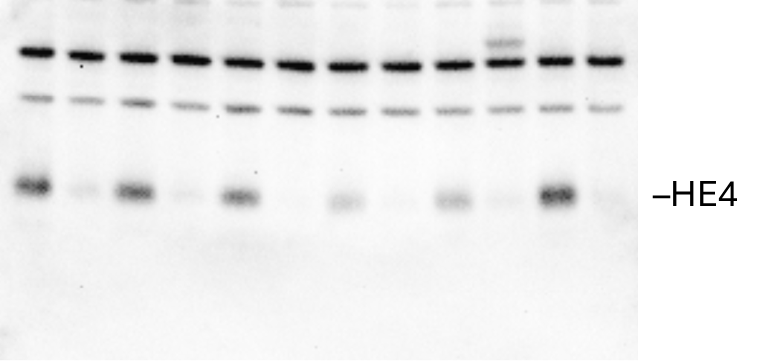


Figure 6C


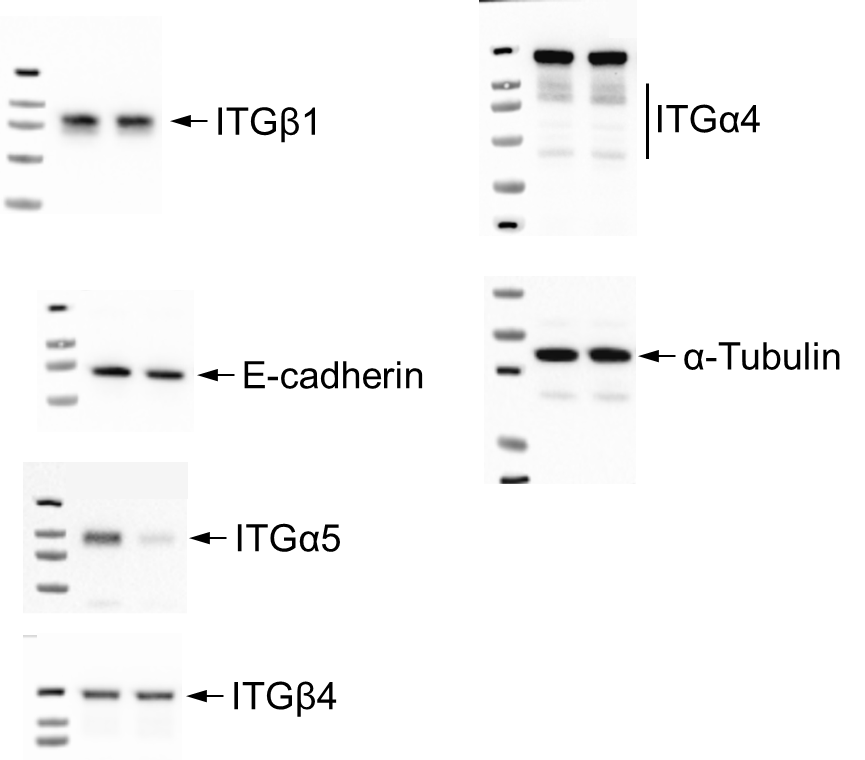


Figure 6D


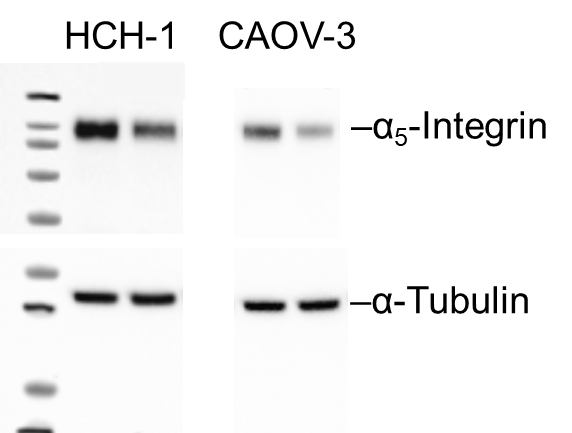


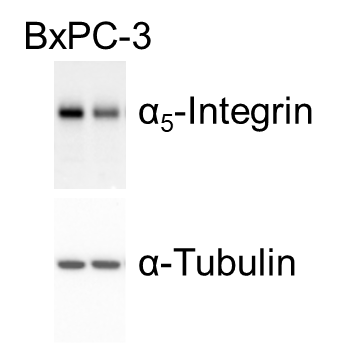

Supplement: S2 File — (DOCX) [file pone.0314564.s002.docx]
